# The role of nature relatedness on environmental ethics awareness and environmental behavior among young adults: a moderated mediation model of environmental organization membership

**DOI:** 10.3389/fpsyg.2026.1750175

**Published:** 2026-02-20

**Authors:** Selman Bolukbasi, Nurullah Yelboga, Yahya Aktu

**Affiliations:** 1 Department of Gerontology, Faculty of Health Sciences, Inonu University, Malatya, Türkiye; 2 Department of Social Work, Faculty of Health Sciences, Sirnak University, Sirnak, Türkiye; 3 Department of Social Service and Consultancy, Eruh Vocational School, Siirt University, Siirt, Türkiye

**Keywords:** environmental behavior, environmental ethics awareness, environmental organization membership, nature relatedness, young adulthood

## Abstract

**Objectives:**

This study seeks to clarify the role of environmental ethics awareness as a mediator and environmental organization membership as a moderator in the relationship between nature relatedness and environmental behavior among young adults.

**Methods:**

The participants consist of 693 young adults (Mage = 22.45 ± 4.37, 60.8% were female). We collected data using a personal information form, the Environmental Ethics Awareness Scale, the Nature Relatedness Scale, and Environmental Behavior Scale. We examined relationships among variables using Pearson's correlation and moderated mediation analysis using Hayes's bootstrapping.

**Results:**

The correlation analyses revealed positive relationships between nature relatedness, environmental ethics awareness, environmental behavior, and environmental organization membership. Environmental ethics awareness mediates the correlation between nature relatedness and environmental behavior. The mediational relationship was further conditioned by environmental organization membership.

**Conclusions:**

The findings highlight how both personal connection to nature and ethical awareness are related to environmental behavior, with these associations being more pronounced among organization members.

## Introduction

Environmental issues have become one of the most pressing concerns for contemporary societies. The risks associated with biodiversity loss, natural resource depletion, and global climate crises are now recognized as universal challenges (Li et al., 2022). Additionally, climate change has emerged as one of the most significant sources of anxiety among individuals (Bouman et al., 2020). Furthermore, factors such as soil, air, and water pollution, global warming, and alterations in environmental components (Gümüş and Buluş, 2020), along with erosion, deforestation, disruptions in production-consumption balance (Karaca, 2007), and food insecurity (Brophy et al., 2023), pose severe threats to ecosystems. The World Health Organization (2025) reports that nearly 99% of the global population is exposed to air pollution exceeding health standards. This ecological crisis, which exacerbates concerns worldwide, has far-reaching implications (Li et al., 2022). Climate change progressively amplifies the adverse effects of other environmental factors on human life and wellbeing (Bongaarts, 2019), negatively impacting both physical and mental health (Clayton et al., 2021). The phenomenon of eco-anxiety (Brophy et al., 2023), financial losses, and environment-related mental health disorders (Field and Barros, 2014) have become increasingly prevalent. Research literature suggests a correlation between rising temperatures and an increased risk of suicide (Thompson et al., 2023), depression, stress (Palinkas and Wong, 2020), and behavioral and psychological disorders (Wang and Horton, 2015), all of which are exacerbated by climate change. Existing findings indicate that environmental problems profoundly and negatively affect human life and health in various dimensions.

Environmental behavior evolves in response to climate change and environmental challenges. Research indicates that environmental behaviors begin developing in early childhood and continue into early adulthood (Yan et al., 2025). However, studies suggest a decline or stagnation in environmental behaviors between the ages of 13 and 18, followed by an increase in adulthood (Otto et al., 2019). Although younger individuals are generally less aware of climate change and recycle less frequently than older adults (Shin and Kim, 2023), research highlights that their environmental actions are shaped by a combination of intrinsic and extrinsic influences, with young adults demonstrating greater openness to behavioral change (Li et al., 2022). A study conducted with 10,000 children and young people aged 16–25 revealed that feelings of helplessness, anger, guilt, and anxiety were prevalent, negatively impacting their daily lives and leading them to perceive the future as frightening (Hickman et al., 2021). Given that Generation Z constitutes approximately one-third of the global population, with the number of individuals aged 15–24 expected to rise from 1.2 billion today to 1.3 billion by 2030, the environmental behaviors of this demographic are of critical importance. Consequently, young people are positioned at the center of solutions for climate change, environmental pollution, and biodiversity loss, and they are regarded as key actors in environmental sustainability (World Health Organization, 2025). A study on Turkish youth found that environmental issues capture their interest and that environmental behavior dynamics significantly influence their engagement in environmental actions (Iba Gürsoy et al., 2022). Another study reported that students identified species extinction and waste-related environmental pollution as major concerns, with findings indicating that environmental awareness positively impacts environmental attitudes and behaviors (Sancak ITB, 2022). According to the Turkish Statistical Institute (TURKSTAT, 2024), young people aged 15–24 constitute 14.9% of Türkiye's total population of 85.66 million. Additionally, the Türkiye Environmental Indicators Report highlights key environmental challenges, including air and water pollution, waste and noise issues, excessive energy consumption, climate change, biodiversity loss, and the mismanagement of agricultural and aquatic resources (T.R. Ministry of Environment U and CC, 2023). The persistence of global environmental issues within Türkiye, coupled with the country's significant youth population, underscores the necessity of examining environmental behavior within the context of young individuals.

The relationship between nature relatedness and environmental behavior has been widely studied in environmental psychology (Nisbet et al., 2009; Mayer and Frantz, 2004). While prior research confirms that emotional and cognitive bonds with nature promote pro-environmental actions, few studies have examined the mediating role of environmental ethics awareness or the moderating effect of environmental organization membership (Schultz, 2000; Stern, 2000). This study addresses these gaps by integrating all three constructs within the concept of nature relatedness (Nisbet et al., 2009; Nisbet and Zelenski, 2013) framework, proposing that ethics awareness explains how nature relatedness translates into behavior (Restall and Conrad, 2015; Whitburn et al., 2020). Given rising ecological engagement among youth, ethics awareness may act as a protective factor sustaining responsible behavior. Organization membership may further amplify this pathway by reinforcing ethical values and collective identity. Accordingly, the study hypothesized that (1) nature relatedness predicts environmental behavior, (2) ethics awareness mediates this relationship, and (3) organization membership moderates the indirect effect. By testing these dynamics simultaneously, the study offers a more nuanced understanding of the psychological and contextual drivers of environmental engagement.

### Nature relatedness and environmental behavior

Nature relatedness refers to individuals' personal connection with nature, encompassing their relationships with all living organisms and various aspects of the natural world, including non-aesthetic elements (Nisbet et al., 2009). This concept is rooted in cognitive, emotional, and experiential dimensions, influencing individuals' environmental concerns and conservation behaviors (Zelenski and Nisbet, 2014). Research consistently shows that stronger bonds with nature fosters a sense of belonging, reinforcing pro-environmental attitudes and movivating susbainability-oriented actions (Mayer and Frantz, 2004; Whitburn et al., 2020). According to the concept of nature relatedness (Nisbet et al., 2009; Nisbet and Zelenski, 2013), such emotional ties encourage environmentally responsible behaviors (Restall and Conrad, 2015; Beery and Wolf-Watz, 2014), as individuals with heightened nature relatedness demonstrate graater awareness and engagement in conservation efforts.

Environmental behavior encompasses all constructive or destructive actions that impact the environment (Süllü and Engin, 2024). It is shaped by external factors, personality traits, and individuals' relationships with nature (Krajhanzl, 2010). Empirical studies highlight that young adults, despite lower climate awareness compared to older cohorts, exhibit a greater propensity for behavioral change when motivated by environmental awareness (Li et al., 2022). Experimental research has shown that students significantly reduce energy consumption and greenhouse gas emissions when motivated by environmental awareness (Cornelius et al., 2014). Similarly, students have adopted climate-friendly dietary habits as a strategy to mitigate climate change (de Boer et al., 2016). Comparative studies highlight that students with higher environmental awareness exhibit stronger pro-environmental intentions (Teixeira et al., 2023). In Türkiye, research has demonstrated that nature relatedness has a significant positive effect on pro-environmental behavior (Nisbet et al., 2009; Nisbet and Zelenski, 2013).

Nature relatedness, encompassing emotional and cognitive dimensions, plays a crucial role in shaping individuals' environmental behaviors, ecological sensitivity, and values (Akçakese et al., 2024; Huang et al., 2022). Closely tied to this is environmental ethics awareness, which involves recognizing and respecting all aspects of the environment and influences pro-environmental attitudes and actions (Kiliç, 2008; Sargin and Dursun, 2024). Research shows that personal moral norms and environmental values significantly predict sustainable behaviors, especially under perceived environmental threats (Stern, 2000; Li et al., 2022). In Türkiye, studies confirm that nature relatedness predicts pro-environmental behavior and strengthens environmental values (Teixeira et al., 2023; Dutcher et al., 2007). According to the concept of nature relatedness (Nisbet et al., 2009; Nisbet and Zelenski, 2013), individuals with a strong bond to nature are more likely to adopt environmentally responsible behaviors, guided by internalized moral norms and values (Restall and Conrad, 2015; Whitburn et al., 2020). With this in mind, the following hypothesis was formulated:

H_1_: *Nature relatedness positively predicts environmental behavior*.

### Environmental ethics awareness as a mediatoring variable

Environmental ethics awareness, from an ecocentric perspective, is the capacity to perceive and evaluate nature's intrinsic value beyond human utility, recognizing the moral worth of all ecological components (Rülke et al., 2020). Such awareness frames environmental issues as ethical imperatives, fostering sustainable behaviors and nature-aligned lifestyles through cognitive, emotional, and normative bonds with the natural world. It further cultivates a positive environmental consciousness, which in turn influences human behavior (Sargin and Dursun, 2024). The definition enviromental ethics awarenness is fragmented, overlapping with concepts like ecological values and biospheric concern. Individuals with a high level of environmental ethics awareness tend to exhibit greater concern for environmental protection and adopt more conservation-oriented attitudes (Karaca, 2007). According to the concept of nature relatedness (Nisbet et al., 2009; Nisbet and Zelenski, 2013), individuals perceive themselves as integral components of nature and acknowledge their reciprocal relationship with the natural world (Restall and Conrad, 2015; Marchetti et al., 2024). The sense of connection to nature reinforces commitment to biospheric values, thereby motivating pro-environmental behaviors (Martin and Czellar, 2017). Specifically, enviromental ethics awarenness may serve as a psychological bridge between nature connectedness and pro-environmental behaviors by activating moral identity processes and normative beliefs. Individuals who feel a moral obligation to protect nature are more likely to act on their awareness. This aligns with the concept of nature relatedness (Nisbet et al., 2009; Nisbet and Zelenski, 2013), which emphasizes that individuals who feel a moral obligation toward nature are more likely to engage in pro-environmental actions.

Empirical research has identified a positive correlation between environmental ethics awareness and sustainable environmental attitudes (Sargin and Dursun, 2024), as well as a significant association with actual pro-environmental behaviors (Buzlukçu, 2023). Buzlukçu (2023) demonstrated that environmental ethics awareness is significantly associated with pro-environmental behavior, with individuals exhibiting higher levels of awareness being more inclined to engage in environmentally friendly actions. Additionally, research has found that nature relatedness and connectedness serve as predictors of pro-environmental behavior (Atik et al., 2023). Thus, both nature relatedness and environmental ethics awareness function as motivational factors for fostering environmentally responsible behavior. Based on these findings, it can be hypothesized that environmental ethics awareness, environmental behavior, and nature relatedness are interconnected, influencing individuals' engagement in sustainable environmental practices. Therefore, the following hypothesis has been developed:

H_2_: *Environmental ethics awareness mediates the nature relatedness-environmental behavior relationship*.

### Environmental organization membership as a moderating variable

Volunteering, defined as engaging in socially beneficial activities without personal gain (Yönten Balaban and Çoban Ince, 2015), plays a vital role in environmental protection. Many individuals join environmental organizations to participate in conservation, advocacy, education, and policy influence efforts (Eryilmaz, 2018). Membership in such organizations enhances environmental awareness, fosters collective action, and promotes responsible behaviors (Pokharel et al., 2025). Studies show that involvement in environmental groups leads to shifts in attitudes and behaviors, stronger pro-environmental intentions, and increased sensitivity to sustainability (Green et al., 2016; Fielding et al., 2008; Aydemir and Alim, 2025; Karahan, 2017; Ogunjinmi et al., 2023). Aligned with the concept of nature relatedness (Nisbet et al., 2009; Nisbet and Zelenski, 2013), organizational membership strengthens individuals' emotional and cognitive bonds with nature, cultivating biospheric values and intrinsic motivation (Restall and Conrad, 2015; Whitburn et al., 2020). This connection fosters ethical awareness and reinforces consistent pro-environmental actions through shared identity and direct engagement with nature. Thus, the following hypothesis was formulated:

H_3_: *Environmental organization membership moderates the relationship between nature relatedness and environmental ethics awareness*.

### The present study

Although prior studies have established a robust relationship between nature relatedness and environmental behavior, little is known about how environmental ethics awareness this relationship or how organizational membership moderates it. Addressing this gap is crucial, as integrating ethical cognition and collective engagement into the concept of nature relatedness (Nisbet et al., 2009; Nisbet and Zelenski, 2013) framework provides a more comprehensive understanding of the psychological and social drivers of pro-environmental behavior. Such an approach not only advances theory but also offers practical insights for designing interventions that foster sustainable actions among youth. Grounded in the concept of nature relatedness (Nisbet et al., 2009; Nisbet and Zelenski, 2013), the study employs a moderated mediation model to investigate these dynamics. According to the concept of nature relatedness (Nisbet et al., 2009; Nisbet and Zelenski, 2013), individuals with a strong psychological bond to nature tend to adopt biospheric values and internalized moral norms, which promote pro-environmental behavior (Mayer and Frantz, 2004; Schultz et al., 2004). Within this framework, environmental ethics awareness may act as a cognitive-moral mediator, reinforcing ethical reasoning and value alignment. Moreover, organizational membership is hypothesized to strengthen this pathway by offering social support, shared identity, and ethical discourse, thereby enhancing the translation of awareness into sustained environmental action.

**Figure 1:**
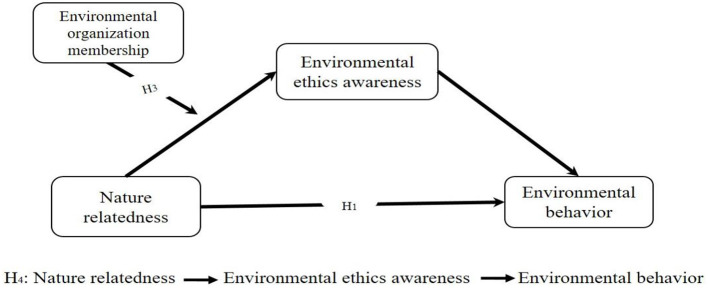
The hypothesized model.

The moderated mediation model assumes a direct relationship between nature relatedness and environmental behavior (H1). In addition, environmental ethics awareness is proposed to mediate this relationship (H2). Finally, environmental organization membership is hypothesized to moderate the indirect effect of nature relatedness on environmental behavior through environmental ethics awareness (H3). Figure 1 presents the hypothesized model.

## Method

### Participants and procedure

This study was conducted with young adults residing in a province within Türkiye's Southeastern Anatolia region, characterized by low socio-economic conditions. A total of 693 participants aged 18–36 were included through convenience sampling, a non-probability technique in which individuals are selected based on their accessibility and willingness to participate (Çokluk et al., 2018). The demographic details of the participants are presented in Table 1.

**Table 1:** Participant demographics.

**Participant demographics Variables**	***n***	**%**	***M***	***SD***
**Gender**				
Female	421	60.8		
Male	272	39.2		
Age			22.32	3.82
**Education level**				
Associated Degree	316	45.6		
Undergraduate	372	53.7		
Graduate	5	0.7		
**Environmental organization membership**				
Yes	94	13.6		
No	599	86.4		

According to Table 1, the majority of participants (60.8%) are female. Participants' ages range between 18 and 36 years, with a mean age of 22.32 years (SD = 3.82). In terms of education, 53.7% hold a university degree.

The study was approved by the Sirnak University Ethics Committee (decision date and number: 22/04/2025 and E-130760) and all procedures complied with ethical standarts for Helsinki Declaration. To ensure procedural transparency and reproducibility, participants were recruited through digital invitations posted on social media groups, university forums, and local youth networks. Those who expressed interest were directed to a Google Forms link, where they first reviewed an informed consent form and then completed the survey. The survey was designed to take approximately 10 min and included attention-check items to ensure data quality. Data collection occurred between May and August 2025. Of the 735 young adults completed the form, 42 were excluded due to incomplete responses or violations normality assuptions, resulting in a final sample of 693 participants. Subsequently, a second ethics approval (2025/24) was obtained from the Inonu University Social and Human Sciences Scientific Research and Publication Ethics Committee.

### Measures

#### Environmental behavior scale

The Environmental Behavior Scale, originally developed by Goldman et al. (2006) and subsequently adapted into Turkish by Timur and Yilmaz (2013), consists of 20 items categorized into six dimensions: resource conservation activities for personal economic benefit, environmentally conscious consumer behavior, nature-related leisure activities, recycling efforts, responsible citizenship, and environmental activism. The scale employs a 5-point Likert measurement system, ranging from “Never” (1) to “Always” (5). Total scores range between 20 and 100, with higher scores indicating greater environmental sensitivity among adults. In this research, we calculated overall scale scores rather than separate factor scores, thereby capturing a more comprehensive representation of the constructs examined. In this study, we relied on overall scale scores rather than separate factor scores, in line with the original development of the instrument. We found the Cronbach's alpha coefficient of 0.84, showing the scale is reliable.

#### Environmental ethics awareness scale

The Environmental Ethics Awareness Scale, developed by Özer and Keleş (2016), comprises 23 items distributed across four dimensions: definition of environmental ethics, objectives of environmental ethics, reasons for its emergence, and strategies for implementation. The scale utilizes a 5-point Likert system, with responses ranging from “Strongly disagree” (1) to “Strongly agree” (5). Possible scores vary from 23 to 115, with higher scores reflecting heightened environmental ethics awareness. Consistent with the original scale, we employed aggregate scores rather than separate factor scores. This study also confirmed the scale's reliability, yielding a Cronbach's alpha of 0.95.

#### Nature relatedness scale

The Nature Relatedness Scale, developed by Nisbet et al. (2009) and later adapted into Turkish by Çakir et al. (2015), consists of 21 items organized into three dimensions: self-perception, environmental perspective, and direct experience with nature. It follows a 5-point Likert format, where responses range from “Strongly disagree” (1) to “Strongly agree” (5), with total scores ranging from 21 to 105. The scale does not contain reverse-scored items. For the purposes of this study, we used total scale scores to provide a more integrated view of the construct. In this study, the scale demonstrated acceptable reliability, with a Cronbach's alpha of 0.72.

### Statistics analysis

Prior to hypothesis testing, we examined the data for normality, since normal distribution is a critical assumption for the validity of parametric statistical analyses. All variables fell within the acceptable range of ±1.5 for skewness and kurtosis (Tabachnick et al., 2019), confirming normal distribution. Pearson's correlation analysis was conducted to explore bivariate relationships among the study variables. Multicollinearity was assessed using standard thresholds: tolerance values above 0.10, variance inflation factors (VIFs) below 10, and condition indexes under 30 (Çokluk et al., 2018). No multicollinearity issues were detected.

To test the hypothesized moderated mediation model, we employed PROCESS Macro Model 7. To ensure the comprehensiveness of the analysis, all scales were included, as each scale captured distinct dimensions of environmental attitudes and behaviors, thereby allowing the mediation and moderation effects in the model to be tested more reliably. In this model, nature relatedness served as the independent variable (X), environmental ethics awareness as the mediator (M), and environmental behavior as the dependent variable (Y). Environmental organization membership was operationalized as a binary moderator (W), coded as 1 = member and 2 = non-member, and was hypothesized to moderate the path from nature relatedness to environmental ethics awareness. Gender was included as a covariate to control for potential confounding effects. To assess the conditional indirect effects, we conducted bootstrap resampling with 10,000 iterations within a 95% confidence interval (CI). Mediation was considered significant if the CI did not include zero (Hayes, 2018). This approach allowed for robust estimation of the moderated mediation effects and ensured the reliability of the findings. We analyzed the data using IBM SPSS version 27 and Hayes' PROCESS Macro 4.2 package programs.

## Results

### Correlation analyses

The relationships among the variables were assessed using Pearson correlation analysis, and the corresponding descriptive statistics and correlations results are showed in Table 2.

**Table 2:** Descriptive statistics and correlation analyses (*N* = 693).

	**Min**.	**Max**.	**M**	**SD**	**Skewness**	**Kurtosis**	**1**	**2**	**3**
1. Environmental ethics awareness	22.00	110.00	92.87	13.57	−1.00	1.61			
2. Environmental behavior	20.00	100.00	62.06	11.54	−0.06	0.58	0.29^**^		
3. Nature relatedness	21.00	100.00	68.84	8.58	−0.18	2.99	0.28^**^	0.39^**^	
4. Environmental organization membership	1 (28.3%)	2 (71.7%)	–	–	−0.97	−1.07	0.44^**^	0.10^**^	0.10^*^

^**^p < 0.01, ^*^p < 0.05, M, Mean; SD, Standard deviation; 1, member; 2, non-member.

Pearson correlations (Table 2) indicated significant positive associations among nature relatedness, environmental ethics awareness, environmental behavior, and organization membership. Environmental ethics awareness emerged as a central factor, relating nature relatedness and organizational involvement to pro-environmental behavior. Overall, individuals with stronger ethical awareness and connection to nature were more likely to act sustainably and participate in environmental organizations. These findings suggest that individuals with higher ethical awareness and stronger nature relatedness are more likely to engage in pro-environmental behaviors and participate in environmental organizations.

### Moderated mediating analysis

After observing relationships between the study variables, the moderated mediating role of environmental ethics awareness and environmental organization membership in the nature relatedness-environmental behavior relationship was examined. Table 3 presents moderated mediational analysis.

**Table 3:** Moderated mediational analysis of nature relatedness, environmental ethics awareness, and environmental behavior.

**Variables**	**M (Environmental ethics awareness)**						**Y (Environmental behavior)**					
	β	***SE***	***t***	***p***	**LLCI**	**ULCI**	β	***SE***	***t***	***p***	**LLCI**	**ULCI**
X (Nature relatedness)	1.34	0.18	7.32	<0.001	0.98	1.70	0.44	0.04	9.25	<0.001	0.35	0.54
C (Gender)	0.14	1.03	0.14	>0.05	−1.89	2.18	0.36	0.93	0.38	>0.05	−1.48	2.20
M (Environmental ethics awareness)							0.16	0.03	5.39	<0.001	0.10	0.22
W (Environmental organization membership)	12.24	0.10	−5.44	<0.001	10.31	14.16						
X^*^W (Nature relatedness^*^ Environmental organization membership)	−0.58	1.00	0.14	<0.001	−0.79	−0.37						
	*R* = 0.53, *R*^2^ = 0.28						*R* = 0.43, *R*^2^ = 0.18					
	*F*_(4, 688)_ = 68.57, *p* < 0.001						*F*_(3, 689)_ = 51.76, *p* < 0.001					

N = 693, bootstrap sample size = 10,000, SD, standard deviation; SE, standard error; LLCI, lower limit 95% CI; ULCI, upper limit 95% CI; X, independent variable; Y, dependent variable; C, covariate; M, mediator; and W, moderator. ^*^p < 0.05.

As seen in Table 3, the non-standardized direct effect between nature relatedness and environmental ethics awareness was significant (*p* < 0.001). Furthermore, the direct effect of the environmental organization membership was found to be significant (*p* < 0.001). However, the covariates of gender no significant effect on environmental ethics awareness (*p* > 0.05). Moreover, the effect of environmental organization membership on environmental ethics awareness via nature relatedness was found to be significant (*p* < 0.001). Accordingly, it was found to explain 28% of the variance in environmental ethics awareness and is therefore significant [*F*_(4, 688)_ = 68.57, *p* < 0.001].

The non-standardized direct effect between nature relatedness and environmental behavior was determined to be significant (*p* < 0.001). In addition, the effect of environmental ethics awareness on environmental behavior was found to be significant (*p* < 0.001). However, the covariates of gender on environmental behavior was found to not be significant (*p* > 0.05). Thus, it was found to explain 18% of the variance in environmental behavior and thus is significant [*F*_(3, 689)_ = 51.76, *p* < 0.001]. Therefore, H1 and H2 were supported. These results support a moderated mediation framework in which nature relatedness influences environmental behavior both directly and indirectly through environmental ethics awareness, and this pathway is moderated by environmental organization membership.

**Figure 2:**
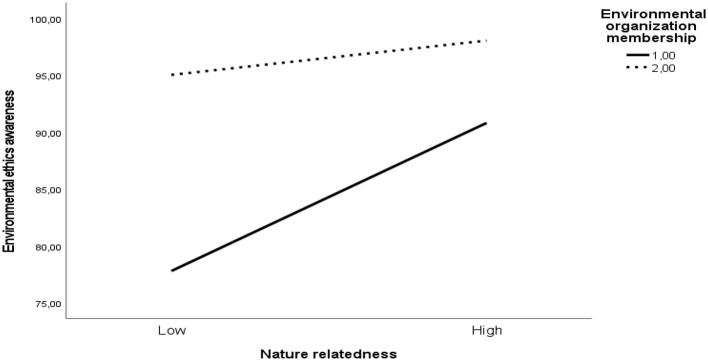
Environmental organization membership's moderating effect on the relationship between nature relatedness and environmental ethics awareness. The solid line represents individuals who are members of environmental organizations, showing a stronger positive association between nature relatedness and ethics awareness. The dotted line represents non-members, for whom the relationship is weaker.

The study analyzed environmental organization membership's moderating role in nature relatedness-environmental behavior relationship through environmental ethics awareness, with Table 4 and Figure 2 presenting the results of the conditional indirect effects of environmental ethics awareness on environmental behavior.

**Table 4:** Conditional indirect effects of environmental organization membership on environmental behavior through environmental ethics awareness.

**Factors**	**Effect**	**SE**	**LLCI**	**ULCI**
Being environmental organization membership (1)	0.12	0.03	0.06	0.19
Not being environmental organization membership (2)	0.03	0.01	0.01	0.05
***Index of moderated mediation***				
Environmental organization membership	−0.10	0.03	−0.16	−0.03

N = 693; bootstrap sample size = 10,000; SD, standard deviation; SE, standard error; LLCI, lower limit 95% CI, and ULCI, upper limit 95% CI.

Table 4 presents the conditional indirect effects of nature relatedness on environmental ethics awareness at different levels of environmental organization membership. Specifically, the analysis examined two levels of the moderator: Level 1 (being a member of an environmental organization) and Level 2 (not being a member). Results indicated that the indirect effect of nature relatedness on environmental behavior via environmental ethics awareness was statistically significant at both levels. For individuals who were members of an environmental organization (Level 1), the effect was stronger (*p* < 0.001). For non-members (Level 2), the effect remained significant but was notably weaker (*p* < 0.001).

As seen in Figure 2 and Table 4, environmental organization membership significantly moderated the indirect effect of nature relatedness on environmental behavior through environmental ethics awareness. The results regarding the mediation index is significant (*p* < 0.001) since the 95% CI does not include zero. The interaction indicates that members exhibit a stronger positive relationship between nature relatedness and environmental ethics awareness, whereas non-members show a weaker association, highlighting the amplifying role of collective engagement. Accordingly, environmental organization membership moderates the relationship between nature relatedness and environmental ethics awareness. Hence, H3 was confirmed.

This pattern is visually supported by the interaction graph, which illustrates a steeper positive slope between nature relatedness and environmental ethics awareness among organization members. In contrast, the slope for non-members is relatively flat, indicating a diminished predictive strength. Overall, the results suggest that organizational involvement amplifies the influence of nature relatedness on ethical awareness, highlighting the role of collective engagement and social context in shaping pro-environmental values and behaviors.

## Discussion

This study investigates the relationship between nature relatedness and environmental behavior among Turkish youth, focusing on the mediating role of environmental ethics awareness and the moderating role of environmental organization membership. To test three hypotheses, both direct and indirect relationships were examined. As a result, all hypotheses in the study were confirmed. The findings indicate that environmental behavior is positively and significantly associated with an increase in nature relatedness. Additionally, environmental ethics awareness mediates the relationship between nature relatedness and environmental behavior. Furthermore, an environmental organization membership moderates the relationship of nature relatedness on environmental behavior through environmental ethics awareness. Accordingly, being an environmental organization membership positively related to environmental behavior via environmental ethics awareness.

This study confirms that nature relatedness positively predicts environmental behavior, consistent with prior research. Meta-analyses and empirical studies have repeatedly demonstrated significant associations between connectedness to nature, environmental responsibility, biospheric values, and pro-environmental actions (Akçakese et al., 2024; Pereira and Forster, 2015; Özdemir, 2019; Mackay and Schmitt, 2019). Collectively, these findings reinforce the theoretical premise of the concept of nature relatedness (Nisbet et al., 2009; Nisbet and Zelenski, 2013), which posits that individuals who perceive themselves as part of the natural world are more inclined to act in its defense (Restall and Conrad, 2015; Schultz et al., 2004). While the relationship is robust across studies, its strength and mechanisms may vary by cultural context. In collectivist societies like Türkiye, nature relatedness may be shaped by communal values and traditional ecological knowledge, enhancing its behavioral influence. This study extends the concept of nature relatedness (Nisbet et al., 2009; Nisbet and Zelenski, 2013) by framing nature relatedness as both a personal disposition and a socially reinforced value system. Future research should examine these dynamics across diverse cultural and socioeconomic settings to build more inclusive models of environmental behavior.

The present study, consistent with the second hypothesis, has found that environmental ethics awareness mediates the relationship between nature relatedness and environmental behavior. Rooted in Wilson's biophilia hypothesis (Wilson, 2002), this relationship reflects how emotional bonds with nature activate cognitive processes that shape values and ethical norms. Prior research within the concept of nature relatedness (Nisbet et al., 2009; Nisbet and Zelenski, 2013) framework indicates that nature relatedness encompasses cognitive, emotional, and behavioral dimensions, each contributing to ecological responsibility (Restall and Conrad, 2015; Schultz et al., 2004). Emotional attachment to nature has been shown to foster pro-environmental attitudes and predict sustainable behaviors (Mayer and Frantz, 2004; Beery and Wolf-Watz, 2014; Mayer et al., 2009), while high levels of ethical awareness promote protective orientations toward the environment (Karaca, 2007; Sargin and Dursun, 2024; Buzlukçu, 2023). Recent studies further suggest that environmental values mediate the link between connectedness to nature and pro-environmental actions (Pereira and Forster, 2015). The mediation effect observed here underscores that nature relatedness alone may not suffice; ethical awareness is necessary to translate attachment into moral action. By integrating moral cognition into the concept of nature relatedness (Nisbet et al., 2009; Nisbet and Zelenski, 2013), this study advances a more comprehensive model that incorporates both affective and normative dimensions of environmental engagement. In culturally specific contexts such as Southeastern Türkiye, where nature is intertwined with heritage and identity, ethical awareness may be shaped by intergenerational narratives and community-based experiences. Accordingly, environmental education should integrate local values, traditional ecological knowledge, and storytelling to cultivate lasting ethical awareness and behavior.

Consistent with the fifth hypothesis, the present study has found that an environmental organization membership moderates the relationship between nature relatedness and environmental ethics awareness. Today, people face increasing pollution, species extinction, and various environmental challenges. The contamination of air, water, and soil with different chemicals poses significant threats to nature. However, it cannot be said that people have completely disconnected from the environment or become indifferent to environmental issues (Nisbet et al., 2009). Young adults are considered an important group in environmental protection, and some studies suggest that they are more environmentally conscious than previous generations (Royne et al., 2011). The literature indicates that an environmental organization membership shapes environmental behaviors and increases individuals' willingness to take action (Pokharel et al., 2025). Students who are members of environmental clubs tend to have higher levels of environmental knowledge and nature relatedness (Ogunjinmi et al., 2023). Participation in environmental activities through club membership has been shown to increase awareness and engagement in conservation efforts (Abdela, 2020). A comparative study found that students who are members of environmental clubs exhibit greater awareness and adopt eco-citizen behaviors more frequently (Houmam and Aomar, 2023). A sense of belonging to nature leads individuals to be more concerned about environmental issues and the future (Schultz et al., 2004). Higher levels of nature relatedness encourage individuals to engage in more pro-environmental behaviors (Davis et al., 2009). These factors not only direct individuals toward environmental organizations but also motivate members of such organizations to adopt more environmentally friendly behaviors. From a theoretical perspective, the concept of nature relatedness (Nisbet et al., 2009; Nisbet and Zelenski, 2013) encompasses emotional, cognitive, and behavioral dimensions, emphasizing individuals' feelings of belonging and attachment to nature (Restall and Conrad, 2015). Nature relatedness fosters concern for nature, while ecological behavior, environmental identity, and biospheric value orientation are fundamental aspects of this theory (Mayer and Frantz, 2004). Thus, similar to previous research findings, an environmental organization membership plays a moderating role in the relationship between nature relatedness and environmental ethics awareness.

Practically, the findings suggest that group-based interventions-such as environmental clubs, youth-led conservation projects, and community participation programs-can significantly strengthen ethical awareness and environmental behavior, especially in regions like Southeastern Türkiye. Here, environmental challenges intersect with strong collectivist values and cultural norms emphasizing social responsibility. Embedding environmental ethics into culturally resonant frameworks-through storytelling, traditional ecological knowledge, and community rituals-may enhance the effectiveness of such interventions. In this context, the study not only confirms key theoretical relationships but also advances the literature by highlighting the interplay between ethical awareness and organizational engagement within specific cultural settings.

## Limitations and future directions

While this study offers valuable insights, several limitations should be noted. The sample consisted of young adults (Sancak ITB, 2022; TURKSTAT, 2024; T.R. Ministry of Environment, Urbanization and Climate Change, 2023; Nisbet et al., 2009; Mayer and Frantz, 2004; Schultz, 2000; Stern, 2000; Nisbet and Zelenski, 2013; Restall and Conrad, 2015; Whitburn et al., 2020; Zelenski and Nisbet, 2014; Beery and Wolf-Watz, 2014; Süllü and Engin, 2024; Krajhanzl, 2010; Cornelius et al., 2014; de Boer et al., 2016; Teixeira et al., 2023; Akçakese et al., 2024; Huang et al., 2022) from Southeastern Türkiye, limiting generalizability due to regional and cultural homogeneity. Convenience sampling and a gender imbalance (60.8% female) further constrain representativeness. The cross-sectional, correlational design prevents causal inference; future longitudinal and experimental studies are recommended. Variables were assessed using total scores, overlooking sub-dimensions that could offer deeper insights. Reliance on self-report measures may introduce social desirability bias, especially in pro-environmental contexts. Future research should incorporate behavioral and implicit tools, and adopt mixed-method approaches to enhance social validity. Additionally, while environmental organization membership was examined as a moderator, other factors like gender, education, and political orientation remain unexplored. Finally, the study tested a unidirectional model; future work should explore bidirectional and alternative pathways using structural equation modeling.

## Conclusions

This study has demonstrated that an environmental organization membership moderates the relationship between nature relatedness and environmental behavior, while environmental ethics awareness plays a mediating role. The findings contribute to the understanding of the association between nature relatedness and environmental behavior, supporting the concept of nature relatedness (Nisbet et al., 2009; Nisbet and Zelenski, 2013), which suggests that both nature relatedness and environmental ethics awareness are related to environmental behavior. These results highlight the significance of nature relatedness, environmental ethics awareness, and an environmental organization membership in shaping environmental behavior. Furthermore, they indicate that nature relatedness and environmental ethics awareness may serve as protective factors for promoting environmental behavior. Addressing environmental ethics awareness and environmental organization membership in therapeutic interventions and volunteer programs for young adults could contribute to the enhancement of environmental behavior. Future research could examine these dynamics across different cultural and age groups, employ longitudinal designs to clarify causal pathways, and integrate qualitative approaches to capture the lived experiences of individuals engaged in environmental organizations.

## Data Availability

The raw data supporting the conclusions of this article will be made available by the authors, without undue reservation.
